# 

**Published:** 1892-12-17

**Authors:** 


.325, Vol. XIII.—Dec. 17th, 1892.

[Registered at the General Post Office as a Newspaper for transmission in the United Kingdom and Abroad.]

WITH EXTRA NURSING SUPPLEMENT
Per Annum, 8s. 6d., post free.
Monthly Parts, 6d.; post free, 8d.
Ditto per Annum; 8s., post free.

### THE HOSPITAL

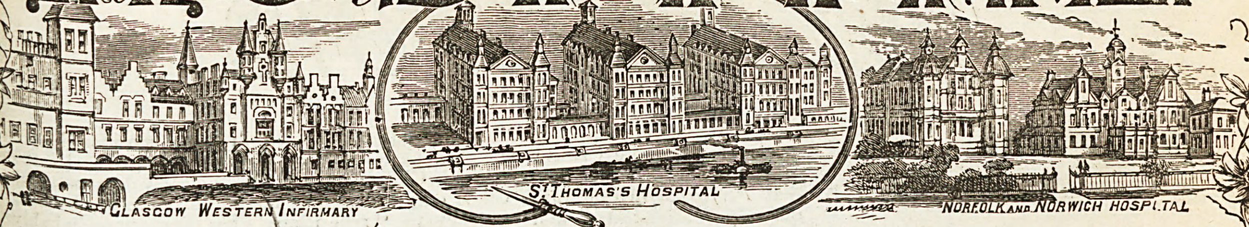

WITH EXTRA NURSING SUPPLEMENT.

No. 325. Vol. XIII.] Saturday, December 17th, 1892. [Twopence.

